# Complete Genomic Sequence and Comparative Analysis of the Genome Segments of Sweet Potato Chlorotic Stunt Virus in China

**DOI:** 10.1371/journal.pone.0106323

**Published:** 2014-08-29

**Authors:** Yanhong Qin, Li Wang, Zhenchen Zhang, Qi Qiao, Desheng Zhang, Yuting Tian, Shuang Wang, Yongjiang Wang, Zhaoling Yan

**Affiliations:** 1 Key Laboratory of Crop Pest Control of Henan Province, Key Laboratory of Pest Management in South of North-China for Ministry of Agriculture of PRC, Institute of Plant Protection, Henan Academy of Agricultural Sciences, Zhengzhou, Henan, China; 2 School of Life Sciences and technology, Nanyang Normal University, Nanyang, Henan, China; 3 Institute of Agricultural Economics and Information, Henan Academy of Agricultural Sciences, Zhengzhou, Henan, China; Institute of Infectious Disease and Molecular Medicine, South Africa

## Abstract

**Background:**

*Sweet potato chlorotic stunt virus* (family *Closteroviridae*, genus *Crinivirus*) features a large bipartite, single-stranded, positive-sense RNA genome. To date, only three complete genomic sequences of SPCSV can be accessed through GenBank. SPCSV was first detected from China in 2011, only partial genomic sequences have been determined in the country. No report on the complete genomic sequence and genome structure of Chinese SPCSV isolates or the genetic relation between isolates from China and other countries is available.

**Methodology/Principal Findings:**

The complete genomic sequences of five isolates from different areas in China were characterized. This study is the first to report the complete genome sequences of SPCSV from whitefly vectors. Genome structure analysis showed that isolates of WA and EA strains from China have the same coding protein as isolates Can181-9 and m2-47, respectively. Twenty *cp* genes and four RNA1 partial segments were sequenced and analyzed, and the nucleotide identities of complete genomic, *cp*, and RNA1 partial sequences were determined. Results indicated high conservation among strains and significant differences between WA and EA strains. Genetic analysis demonstrated that, except for isolates from Guangdong Province, SPCSVs from other areas belong to the WA strain. Genome organization analysis showed that the isolates in this study lack the *p22* gene.

**Conclusions/Significance:**

We presented the complete genome sequences of SPCSV in China. Comparison of nucleotide identities and genome structures between these isolates and previously reported isolates showed slight differences. The nucleotide identities of different SPCSV isolates showed high conservation among strains and significant differences between strains. All nine isolates in this study lacked *p22* gene. WA strains were more extensively distributed than EA strains in China. These data provide important insights into the molecular variation and genomic structure of SPCSV in China as well as genetic relationships among isolates from China and other countries.

## Introduction

Sweet potato (*Ipomoea batatas*) is the third most important root crop after potato and cassava [1], [2]. China is currently the largest producer of sweet potato; the country cultivates the crop over an average of 4.1 million hectares of planting area, which accounts for 48.29% of the worldwide total [3], [4]. Over 30 viruses are known to infect sweet potato [5]. *Sweet potato chlorotic stunt virus* (SPCSV) is the most devastating virus affecting sweet potato. SPCSV, which was previously known as sweet potato sunken vein virus, belongs to genus *Crinivirus* of family *Closteroviridae* [6]–[8]. SPCSV was first reported in the 1970s [9]. It is phloem-limited and transmitted in a semi-persistent manner by whitefly. The virus has the second largest genome and contains a single-stranded bipartite positive-sense RNA genome [10], [11]. SPCSV is often found in co-infection with *Sweet potato feathery mottle virus* (SPFMV), a member of the genus *Potyvirus* that causes a synergistic disease called sweet potato virus disease (SPVD); SPVD is the main viral constraint affecting sweet potatoes worldwide [12]–[14]. Plants with SPVD exhibit severe symptoms, such as leaf strapping, vein clearing, chlorosis, stunting, leaf distortion, and even death, and the disease causes yield losses ranging from 70% to 100% [15]–[18]. Molecular studies have shown that co-infection of SPCSV enhances SPFMV RNA viral titers by at least 600-fold, whereas SPCSV titers remain equal or are reduced compared with single infection [12], [13], [17], [19]. Besides SPFMV, several other viruses belonging to the genera *Potyvirus*, *Carlavirus*, *Cucumovirus*, *Ipovovirus*, and *Cavemovirus* can result in synergistic diseases and severely affect sweet potato yield upon co-infection with SPCSV [18], [20].

SPCSV is distributed worldwide and has been detected in all sweet potato production areas except those in the Pacific region [3], [5], [21], [22]. Based on serological studies, SPCSV can be divided into two distantly related strains: the East African (EA) strain and the West African (WA) strain [23], [24]. A similar subdivision into two genetic strains was revealed by phylogenetic analysis of the coat protein (*cp*) and heat shock protein 70 homolog (*hsp70h*) gene sequences [3], [25]–[27] as well as analysis of the RNA1 sequences [28]. WA strains are more widely distributed than EA strains [27], [29]–[31]. The complete genomic sequence of SPCSV was first determined by Kreuze [11]. As SPCSV has a low titer in sweet potato plants and always co-infects sweet potatoes with other viruses in field [22], [32]–[34], separation and purification of SPCSV is difficult to perform and determination of the SPCSV genomic sequence is greatly constrained. To date, only three SPCSV genomic sequences can be accessed through GenBank, including two complete sequences of the SPCSV EA strain and one sequence of the SPCSV WA strain [11], [29], [35]. Partial sequence analysis shows that not all EA strain isolates include the *p22* open reading frame (ORF) at the 3′ end of RNA1; other isolates may lack a 767 nt region of RNA1 that includes the *p22* gene [29]. The *p22* gene has only been found in isolates from Uganda [28], [29], [36]. Regardless of the presence of *p22*, isolates of SPCSV act synergistically with SPFMV in sweet potato plants and significantly enhance SPFMV titers. However, co-infection of SPFMV with SPCSV isolates containing *p22* causes more severe symptoms in the indicator plant *Ipomoea setosa* than co-infection of SPFMV with SPCSV isolates lacking *p22* [28].

Recent studies have focused on the synergism of SPCSV with other unrelated viruses; the incidence, distribution, and effects of SPVD on sweet potato yield; and management approaches to SPVD [18], [33], [37]. Limited information is available on the genetic variability of SPCSV, and this information is based on analysis of the nucleotide sequences of *hsp70h* and *cp* genes on RNA2 and *p7*, *p22*, and *RNase3* genes on RNA1 [3], [25]–[27], [36].

In China, SPCSV was first detected in 2011 [38], and SPVD was first reported in China in 2012 [39]. Subsequent to its discovery, the virus developed and caused a serious epidemic from 2012 to 2013 [27]. Despite the importance of the virus, however, knowledge of the molecular characterization and genetic diversity of SPCSV in China, which is an important sweet potato production area, remains limited. Moreover, the presence or absence of the *p22* gene in China isolates is unclear.

The current study presents the complete genome sequences of five SPCSV EA and WA isolates obtained from different areas in China. This study is the first to report the complete genome sequences of SPCSV from whitefly vectors, and we provide a simplified and convenient method for cloning SPCSV genomes. The genomic structures of the five isolates were analyzed and compared with three isolate genomes obtained from GenBank. Nucleotide and amino acid identities, including the molecular variance of different isolates, were also compared and a phylogenetic tree was constructed. To confirm the genetic variability of the *cp* gene of WA strains and investigate whether or not the *p22* gene is present in China, we also described the *cp* gene sequences of 20 other isolates and 4 genome segments of RNA1 flanking the *p22* insertion region. Genetic variability and phylogenetic relationships among these isolates were further analyzed.

## Results

### Complete nucleotide sequence analyses of SPCSV from China and comparison with other isolates

**Figure 1:**
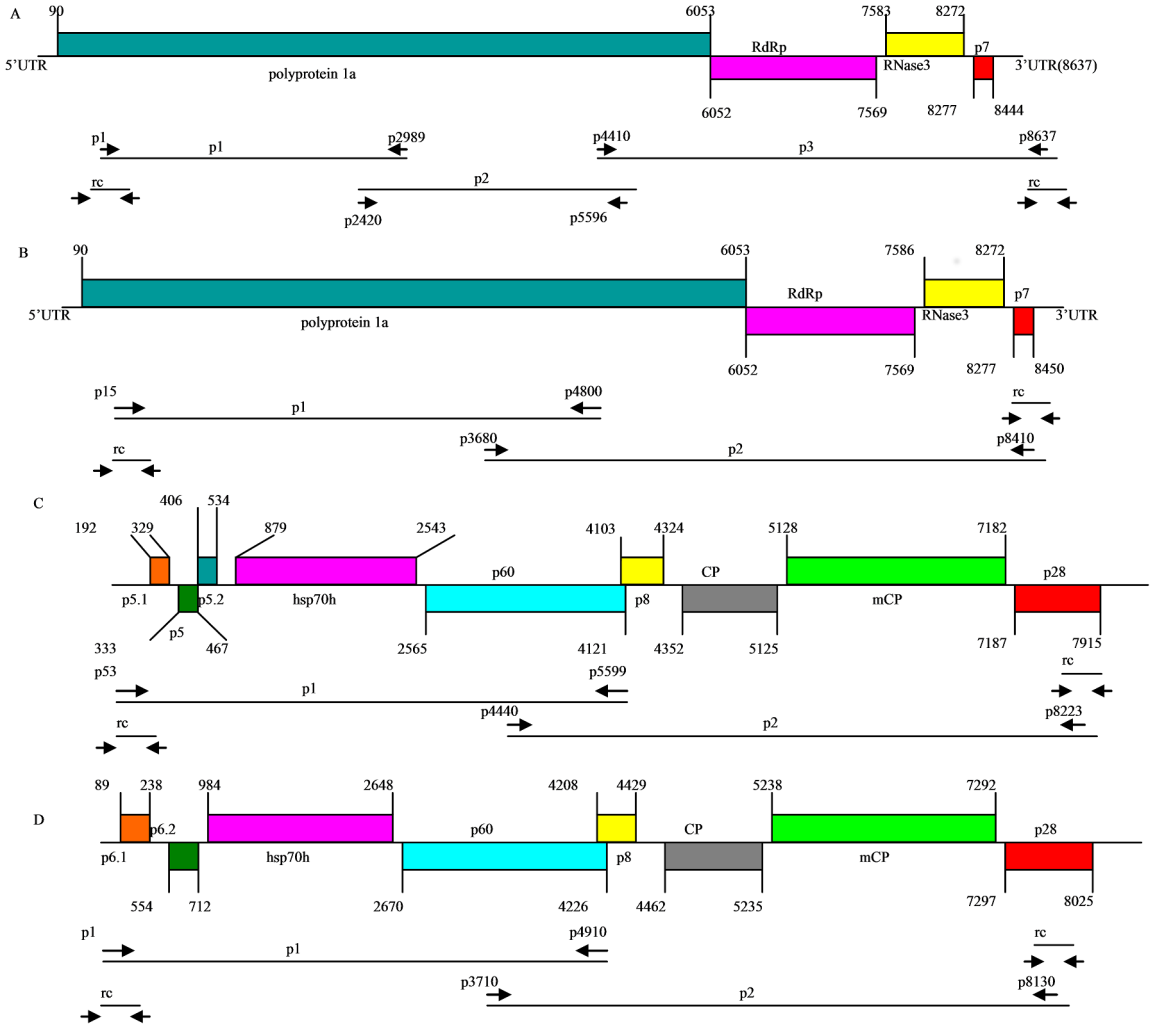
Schematic diagram of genomic organization and genome cloning strategy of SPCSV. Solid lines represent RNA genomes and boxes represent ORFs. Putative protein products are indicated. p1, p2, and p3 represent RT-PCR-generated sequences using specific primers. rc represents the 5- and 3-terminal clones generated by 5′ and 3′ RACE, respectively. Primer sequences are shown in Table 2. (A) Schematic diagram of the genomic organization and genome cloning strategy of the SPCSV WA strain RNA1 segment. (B) Schematic diagram of the genomic organization and genome cloning strategy of the SPCSV EA strain RNA1 segment. (C) Schematic diagram of the genomic organization and genome cloning strategy of the SPCSV WA strain RNA2 segment. (D) Schematic diagram of the genomic organization and genome cloning strategy of the SPCSV EA strain RNA2 segment.

The complete genomic sequences of five isolates of SPCSV belonging to WA and EA strains from different areas in China were characterized by RT-PCR using viruliferous whitefly as materials. The 5′ and 3′ UTRs of virus genome segments RNA1 and RNA2 were also determined by rapid amplification of cDNA ends (RACE). The cloning strategy and a schematic representation of the SPCSV genome organization are shown in Figure 1. The complete genome nucleotide sequences of the five SPCSV isolates were deposited in GenBank under accession numbers KC146840–KC146843 and KC888961–KC888966 (Table 1). Table 2 shows the primer sequences and their corresponding positions in the SPCSV genome.

**Table 1:** Name, strain assignment, geographic origin, segment length, reference, and GenBank accession number of all isolates or samples used in this study.

Isolate names	accession numbers	Serotype	Segments	Segment length (bp)	Geographical origin	Reference
Jiangsu-2011	KC146840	WA	RNA1	8637	Jiangsu	This study
Jiangsu-2011	KC146841	WA	RNA2	8107	Jiangsu	This study
Guangdong-2011	KC146842	EA	RNA1	8622	Guangdong	This study
Guangdong-2011	KC146843	EA	RNA2	8217	Guangdong	This study
Sichuan-12-8	KC888964	WA	RNA1	8637	Sichuan	This study
Sichuan-12-8	KC888961	WA	RNA2	8107	Sichuan	This study
Sichuan-12-12	KC888965	WA	RNA1	8637	Sichuan	This study
Sichuan-12-12	KC888962	WA	RNA2	8107	Sichuan	This study
Chongqing-12-8	KC888966	WA	RNA1	8637	Chongqing	This study
Chongqing-12-8	KC888963	WA	RNA2	8107	Chongqing	This study
m2-47	HQ291259	EA	RNA1	8637	Peru	Cuellar et al. 2011
m2-47	HQ291260	EA	RNA2	8219	Peru	Cuellar et al. 2011
Uganda	AJ428554	EA	RNA1	9407	Uganda	Kreuze et al. 2002
Uganda	AJ428555	EA	RNA2	8223	Uganda	Kreuze et al. 2002
Can181-9	FJ807784	WA	RNA1	8637	Spain	Trenado et al. 2012
Can181-9	FJ807785	WA	RNA2	8108	Spain	Trenado et al. 2012
Jiangsu-11-19	KC243096	WA	RNA1	1356	Jiangsu	This study
Anhui-11-2	KC243097	WA	RNA1	1356	Anhui	This study
Chongqing-11-8	KC243098	WA	RNA1	1356	Chongqing	This study
Zhejiang-11-4	KC243099	EA	RNA1	1356	Zhejiang	This study
Sichuan-11-28	KC243079	WA	RNA2	774	Sichuan	This study
Jiangxi-11-3	KC243080	WA	RNA2	774	Jiangxi	This study
Guangxi-11-5	KC243081	WA	RNA2	774	Guangxi	This study
Guangxi-11-7	KC243082	WA	RNA2	774	Guangxi	This study
Hebei-11-5	KC243083	WA	RNA2	774	Hebei	This study
Shanxi-11-6	KC243084	WA	RNA2	774	Shanxi	This study
Shandong-11-7	KC243085	WA	RNA2	774	Shandong	This study
Shaanxi-11-1	KC243086	WA	RNA2	774	Shaanxi	This study
Jiangsu-11-17	KC243087	WA	RNA2	774	Jiangsu	This study
Jiangsu-11-19	KC243088	WA	RNA2	774	Jiangsu	This study
Anhui-11-2	KC243089	WA	RNA2	774	Anhui	This study
Hunan-11-4	KC243090	WA	RNA2	774	Hunan	This study
Chongqing-11-3	KC243091	WA	RNA2	774	Chongqing	This study
Henan-11-28	KC243092	WA	RNA2	774	Henan	This study
Guangdong-11-9	KC243093	WA	RNA2	774	Guangdong	This study
Zhejiang-11-3	KC243094	WA	RNA2	774	Zhejiang	This study
Zhejiang-11-4	KC243095	WA	RNA2	774	Zhejiang	This study
Guangdong-2009	HM773432	EA	RNA2	774	Guangdong	Qiao et al. 2011
Sichuan-10-77	KC146844	WA	RNA2	774	Sichuan	This study

**Table 2:** Polymerase chain reaction primers and PCR conditions used to amplify the SPCSV genome, CP gene, and RNA1 partial sequences.

Primer name	Sequence (5′ to 3′)	Nucleotide position	Size of amplificons (bp)	polarity	PCR conditions
WA-RNA1-1	GAAATACTTCCAGCTATCCAAATTTGGTG	1–29	2989	Sense	94°C, 3 minutes, 1 cycle; 94°C, 30 seconds, 55°C, 30 seconds,
WA-RNA1-2989	ATACGTCTCTCTCCAACGACAAC	2967–2989		Antisense	72°C, 3 minutes, 30 cycles; 72°C, 10 minutes, 1 cycle
WA-RNA1-2420	AGAGAACAACTTCATTTCTACTCAATTGT	2421–2449	3205	Sense	94°C, 3 minutes, 1 cycle; 94°C, 30 seconds, 55°C, 30 seconds,
WA-RNA1-5596	GATAAATAAGTTTACCTGTATTGTCGGTC	5597–5625		Antisense	72°C, 3.5 minutes, 30 cycles; 72°C, 10 minutes, 1 cycle
WA-RNA1-4410	GAGCATCAACTGTGGACGGCGAACCAAGC	4411–4439	4227	Sense	94°C, 3 minutes, 1 cycle; 94°C, 30 seconds, 55°C, 30 seconds,
WA-RNA1-8637	AACCTAGTTATTTAAATACTAGGTTTTCC	8609–8637		Antisense	72°C, 4.5 minutes, 30 cycles; 72°C, 10 minutes, 1 cycle
WA-RNA2-53	CATTGGTTGTCGTCATGACTCGCAT	53–77	5574	Sense	94°C, 3 minutes, 1 cycle; 94°C, 30 seconds, 55°C, 30 seconds,
WA-RNA2-5599	CCAACTTACCAGATTTCGAGAACTGTAC	5599–5626		Antisense	72°C, 6 minutes, 30 cycles; 72°C, 10 minutes, 1 cycle
WA-RNA2-4440	ATGCGTCTCGTCGTGACGTCCAGACTG	4440–4466	3668	Sense	94°C, 3 minutes, 1 cycle; 94°C, 30 seconds, 55°C, 30 seconds,
WA-RNA2-8223	GGCCTAGTTATTTAAATACTAGGTTTTCC	8079–8107		Antisense	72°C, 4 minutes, 30 cycles; 72°C, 10 minutes, 1 cycle
EA-RNA1-15	TATCCAAATTTGGTGTGTTCTGCAG	15–39	4815	Sense	94°C, 3 minutes, 1 cycle; 94°C, 30 seconds, 55°C, 30 seconds,
EA-RNA1-4800	TTCCAATTGTGATAGATAAAGATCTACC	4802–4829		Antisense	72°C, 5 minutes, 30 cycles; 72°C, 10 minutes, 1 cycle
EA-RNA1-3680	GGTGAGTAAGAAGGATAAATTGATCTCG	3680–3707	4754	Sense	94°C, 3 minutes, 1 cycle; 94°C, 30 seconds, 55°C, 30 seconds,
EA-RNA1-8410	TTCTAATACTCAAAAGGCAATATACAAAC	8405–8433		Antisense	72°C, 5 minutes, 30 cycles; 72°C, 10 minutes, 1 cycle
EA-RNA2-1	GAAATACTACCCAGGTTTTTCCATGAGT	1–28	4933	Sense	94°C, 3 minutes, 1 cycle; 94°C, 30 seconds, 55°C, 30 seconds,
EA-RNA2-4910	GCATGTGATTGATGAAACTATGAGTTC	4907–4933		Antisense	72°C, 5 minutes, 30 cycles; 72°C, 10 minutes, 1 cycle
EA-RNA2-3710	TTACCTAGGGACGTTGACGAATTGGT	3705–3730	4452	Sense	94°C, 3 minutes, 1 cycle; 94°C, 30 seconds, 55°C, 30 seconds,
EA-RNA2-8130	TTCATACACACACTCTAAATAGAAATACG	8128–8156		Antisense	72°C, 4.5 minutes, 30 cycles; 72°C, 10 minutes, 1 cycle
RdRp-F	CAANACNAANGAATTGAACAT	7176–7196	1356 or 2081	Sense	94°C, 3 minutes, 1 cycle; 94°C, 30 seconds, 53°C, 30 seconds,
SVV-R3	TTTTTGAGNTTTTANAATACACAC	8508–8531		Antisense	72°C, 2 minutes, 30 cycles; 72°C, 10 minutes, 1 cycle
CP-F	ATGGCTGATAGCACTAAAGTCGA	4352–4374	774	Sense	94°C, 3 minutes, 1 cycle; 94°C, 30 seconds, 58°C, 30 seconds,
CP-R	TCAACAGTGAAGACCTGTTCCAG	5103–5125		Antisense	72°C, 45 seconds, 30 cycles; 72°C, 10 minutes, 1 cycle
Hsp70h-F	AGTGGTGAYGTAATAGTCGGTGG	1008–1030	365	Sense	94°C, 3 minutes, 1 cycle; 94°C, 30 seconds, 58°C, 30 seconds,
Hsp70h-R	GCTAACGATTCACADACAGACTTCA	1348–1372		Antisense	72°C, 30 seconds, 30 cycles; 72°C, 10 minutes, 1 cycle

The nucleotide sequences of the Guangdong-2011 isolate genome RNA1 and RNA2 comprised 8622 and 8217 bp, respectively. The genome segment RNA1 of Guangdong-2011 isolate had an 89 nt 5′-UTR, a 172 nt 3′-UTR, and four ORFs: nucleotides 90–6053 (*p227*), 6052–7569 (*RdRp*), 7586–8272 (*RNase3*), and 8277–8450 (*p7*). The genome segment RNA2 had an 88 nt 5′-UTR, a 192 nt 3′-UTR, and contained eight ORFs: nucleotides 89–238 (*p6.1*), 554–712 (*p6.2*), 984–2648 (*hsp70h*), 2670–4226 (*p60*), 4208–4429 (*p8*), 4462–5235 (major *cp*), 5238–7292 (minor *cp*), and 7297–8025 (*p28*). The nucleotide sequences of the Jiangsu-2011, Sichuan-12-8, Sichuan-12-12, and Chongqing-12-8 isolate genome RNA1 and RNA2 comprised 8637 and 8107 bp, respectively. The genomic segment RNA1 of these isolates had an 89 nt 5′-UTR, a 193 nt 3′-UTR, and contained four ORFs: nucleotides 90–6053 (*1a*), 6052–7569 (*RdRp*), 7583–8272 (*RNase3*), and 8277–8444 (*p7*). The genomic segment RNA2 of these isolates had a 191 nt 5′-UTR, a 192 nt 3′-UTR, and contained nine ORFs: nucleotides 192–329 (*p5.2*), 333–467 (*p5*), 406–534 (*p5.1*), 879–2543 (*hsp70h*), 2565–4121 (*p60*), 4103–4324 (*p8*), 4352–5125 (major *cp*), 5128–7182 (minor *cp*), and 7187–7915 (*p28*). Comparisons of the genomic structures of RNA1 and RNA2 between this study and the reported isolates are shown in Tables 3 and 4.

**Table 3:** Comparison of genomic structures of RNA1.

	Can181-9	Chongqing-12-8	Jiangsu-2011	Sichuan-12-8	Sichuan-12-12	Guangdong-2011	m2-47	Uganda
5′UTR	1–89	1–89	1–89	1–89	1–89	1–89	1–89	1–89
polyprotein 1a	90–6053	90–6053	90–6053	90–6053	90–6053	90–6053	90–6053	90–6053
RdRP	6052–7569	6052–7569	6052–7569	6052–7569	6052–7569	6052–7569	6004–7569	6052–7569
RNase3	7583–8272	7583–8272	7583–8272	7583–8272	7583–8272	7586–8272	7586–8272	7586–8272
p7	8277–8444	8277–8444	8277–8444	8277–8444	8277–8444	8277–8450	8277–8450	8277–8450
p22								8606–9181
3′UTR	8445–8637	8445–8637	8445–8637	8445–8637	8445–8637	8451–8622	8451–8637	9182–9407

**Table 4:** Comparison of genomic structures of RNA2.

	Can181-9	Chongqing-12-8	Jiangsu-2011	Sichuan-12-8	Sichuan-12-12	Guangdong-2011	m2-47	Uganda
5′UTR	1–191	1–191	1–191	1–191	1–191	1–88	1–90	1–90
p5.2/p6.1	192–329	192–329	192–329	192–329	192–329	89–238	91–240	91–240
p5	333–467	333–467	333–467	333–467	333–467			
p5.1/p6.2	406–534	406–534	406–534	406–534	406–534	554–712	556–714	
hsp70h	880–2544	879–2543	879–2543	879–2543	879–2543	984–2648	986–2650	987–2651
p60	2566–4122	2565–4121	2565–4121	2565–4121	2565–4121	2670–4226	2672–4228	2673–4229
p8	4104–4325	4103–4324	4103–4324	4103–4324	4103–4324	4208–4429	4210–4431	4211–4432
CP	4353–5126	4352–5125	4352–5125	4352–5125	4352–5125	4462–5235	4464–5237	4465–5241
mCP	5129–7183	5128–7182	5128–7182	5128–7182	5128–7182	5238–7292	5240–7294	5244–7298
p28	7188–7916	7187–7915	7187–7915	7187–7915	7187–7915	7297–8025	7299–8027	7303–8031
3′UTR	7917–8108	7916–8107	7916–8107	7916–8107	7916–8107	8026–8217	8028–8217	8032–8223

Multiple sequence comparisons showed that the RNA1 sequence of the Guangdong-2011 isolate was similar to that of the SPCSV EA strain. RNA1 and RNA2 genome segments of the Guangdong-2011 isolate were 99.27% and 99.68% identical to that of the EA m2-47 isolate and 82.44% and 69.99% identical to that of the WA Can181-9 isolate, respectively. The Jiangsu-2011, Sichuan-12-8, Sichuan-12-12, and Chongqing-12-8 isolates showed a closer genetic relationship to the WA Can181-9 isolate than to the EA m2-47 isolate. The nucleotide sequence identities of RNA1 and RNA2 segments respectively ranged from 98.90% to 99.26% and from 98.80% to 99.17% when these four isolates were compared with the Can181-9 isolate of the WA strain. By contrast, the nucleotide sequence identities of RNA1 and RNA2 segments respectively ranged from 82.33% to 82.41% and from 69.80% to 69.98% when these four isolates were compared with the m2-47 isolate of the EA strain.

The pairwise percent identity of complete genomic sequences of the five isolates in China and three isolates retrieved from GenBank was calculated in multiple alignment (Table 5). Results showed that the RNA1 and RNA2 segment nt sequence identities of all isolates ranged from 81.5% to 99.6% and from 70.9% to 99.7%, respectively. The nt sequence identities in the WA strain were 98.8%–99.6% for RNA1 and 98.8%–99.7% for RNA2. The nt sequence identities in EA strains were 97.5%–99.4% for RNA1 and 98.3%–99.7% for RNA2. The nt identities between WA and EA strains were 81.5%–82.7% for RNA1 and 70.9%–71.2% for RNA2. This study revealed that the complete genomic sequences of the same strain group display a high degree of conservation. Compared with the reported sequences, the Chinese isolates showed high conservation and low molecular variation in their genomic sequences.

**Table 5:** Analysis of nucleotide sequence identities (%) of RNA1 (lower diagonal) and RNA2 (upper diagonal) segments of SPCSV isolates in this study and those retrieved from the database.

Isolates	1	2	3	4	5	6	7	8
1. Can181-9		99.0	98.8	99.2	99.2	71.0	70.9	71.0
2. Chongqing-12-8	98.9		99.1	99.1	99.1	71.0	70.9	71.0
3. Jiangsu-2011	98.9	99.6		98.8	98.8	71.1	71.0	71.2
4. Sichuan-12-8	99.3	98.8	98.8		99.7	71.0	70.9	71.1
5. Sichuan-12-12	99.3	98.8	98.8	99.6		71.0	70.9	71.0
6. Guangdong-2011	82.6	82.5	82.5	82.5	82.6		99.7	98.3
7. m2-47	82.7	82.6	82.5	82.6	82.6	99.4		98.4
8. Uganda	81.6	81.5	81.5	81.6	81.6	97.5	97.6	

**Figure 2:**
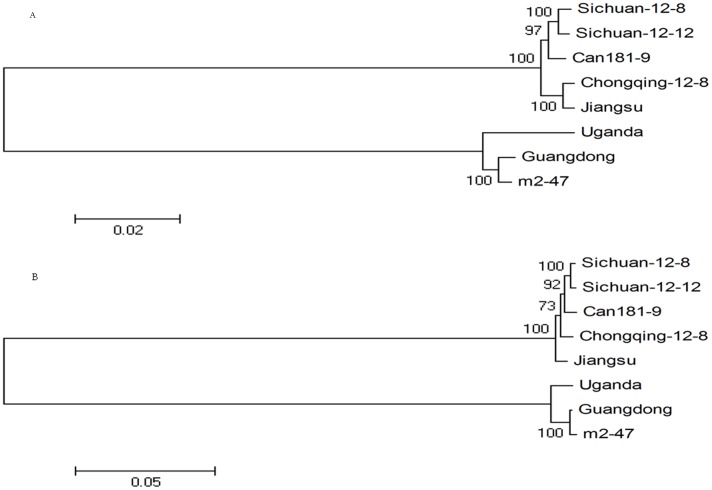
Phylogenetic analysis of genomic RNA1 (A) and RNA2 (B) segments of five isolates determined in China and three isolates retrieved from GenBank. Neighbor-joining trees were constructed via the maximum composite likelihood substitution model using MEGA (version 4.0). Numbers at branches represent bootstrap values of 1000 replicates. The scale bar shows the number of nucleotide substitutions per site.

MEGA 4.0 was used to construct a phylogenetic tree of the five complete genomic sequences determined in this study and three isolates obtained from GenBank (Figure 2). Results indicated that Jiangsu-2011, Sichuan-12-8, Sichuan-12-12, and Chongqing-12-8 belong to the same branch as the Can181-9 isolate while Guangdong-2011 belongs to the same branch as the m2-47 and Uganda isolates.

### Comparison of the length and nucleotide identity of the 5′ and 3′ UTRs of RNA1 and RNA2

Analysis of RNA1 5′ UTRs indicated 89 nt in Guangdong-2011, m2-47, Uganda (EA strain), Jiangsu-2011, Sichuan-12-8, Sichuan-12-12, Chongqing-12-8 and Can181-9 (WA strain), a nucleotide identity of over 98% between different isolates in the WA and EA strains, and a nucleotide identity of 85.4%–87.6% between WA and EA strains (Table 6).

**Table 6:** Nucleotide (lower diagonal) and amino acid (upper diagonal) (%) sequence identities of UTRs, polyprotein 1a (p227), RdRp, RNase3, p7, hsp70h, p60, p8, CP, mCP, and p28 among SPCSV isolates.

	Can181-9	Chongqing-12-8	Jiangsu-2011	Sichuan-12-8	Sichuan-12-12	Guangdong-2011	m2-47	Uganda
**RNA1-5′UTR**								
Can181-9								
Chongqing-12-8	98.9							
Jiangsu-2011	97.8	98.9						
Sichuan-12-8	98.9	100	98.9					
Sichuan-12-12	98.9	100	98.9	100				
Guangdong-2011	85.4	86.5	87.6	86.5	86.5			
m2-47	85.4	86.5	87.6	86.5	86.5	100		
Uganda	85.4	86.5	87.6	86.5	86.5	98.9	98.9	
**polyprotein 1a**	Can181-9	Chongqing-12-8	Jiangsu-2011	Sichuan-12-8	Sichuan-12-12	Guangdong-2011	m2-47	Uganda
Can181-9		98.8	98.7	99.3	99.5	87.2	87.4	87.5
Chongqing-12-8	99.0		99.2	98.7	98.9	87.3	87.5	87.6
Jiangsu-2011	98.9	99.6		98.6	98.8	87.4	87.6	87.6
Sichuan-12-8	99.3	98.8	98.7		99.5	87.2	87.4	87.5
Sichuan-12-12	99.3	98.8	98.7	99.5		87.3	87.5	87.6
Guangdong-2011	81.2	81.2	81.0	81.2	81.2		98.7	97.6
m2-47	81.3	81.3	81.1	81.2	81.3	99.4		98.0
Uganda	81.3	81.2	81.1	81.2	81.3	98.3	98.6	
**RdRp**	Can181-9	Chongqing-12-8	Jiangsu-2011	Sichuan-12-8	Sichuan-12-12	Guangdong-2011	m2-47	Uganda
Can181-9		99.2	99.6	99.4	99.2	96.2	96.4	96.6
Chongqing-12-8	99.1		99.6	99.0	98.8	96.0	96.2	96.4
Jiangsu-2011	99.3	99.8		99.4	99.2	96.2	96.4	96.6
Sichuan-12-8	99.4	99.1	99.3		99.4	96.0	96.2	96.4
Sichuan-12-12	99.4	99.1	99.3	99.7		95.8	96.0	96.2
Guangdong-2011	87.6	87.4	87.5	87.4	87.4		99.2	99.4
m2-47	87.6	87.4	87.5	87.4	87.4	99.5		99.8
Uganda	87.7	87.5	87.5	87.4	87.4	99.4	99.6	
**RNase3**	Can181-9	Chongqing-12-8	Jiangsu-2011	Sichuan-12-8	Sichuan-12-12	Guangdong-2011	m2-47	Uganda
Can181-9		97.4	97.8	98.3	97.8	80.7	79.8	81.1
Chongqing-12-8	98.3		99.6	97.8	97.4	80.7	79.8	81.1
Jiangsu-2011	98.8	99.4		98.3	97.8	81.1	80.3	81.6
Sichuan-12-8	98.8	98.6	99.1		98.7	81.1	80.3	81.6
Sichuan-12-12	98.7	98.4	99.0	99.6		80.7	79.8	81.1
Guangdong-2011	83.0	82.8	83.4	83.3	83.1		99.1	99.1
m2-47	82.8	82.7	83.3	83.1	83.0	99.4		98.2
Uganda	83.1	83.0	83.6	83.4	83.3	99.1	98.7	
**p7**	Can181-9	Chongqing-12-8	Jiangsu-2011	Sichuan-12-8	Sichuan-12-12	Guangdong-2011	m2-47	Uganda
Can181-9		98.2	98.2	98.2	100	63.6	63.6	61.8
Chongqing-12-8	98.8		100	96.4	98.2	63.6	63.6	61.8
Jiangsu-2011	98.8	100		96.4	98.2	63.6	63.6	61.8
Sichuan-12-8	99.4	98.2	98.2		98.2	61.8	61.8	60.0
Sichuan-12-12	100	98.8	98.8	99.4		63.6	63.6	61.8
Guangdong-2011	78.0	76.8	76.8	77.4	78.0		100	98.2
m2-47	78.0	76.8	76.8	77.4	78.0	100		98.2
Uganda	76.2	75.0	75.0	75.6	76.2	98.3	98.3	
**RNA1-3′UTR**	Can181-9	Chongqing-12-8	Jiangsu-2011	Sichuan-12-8	Sichuan-12-12	Guangdong-2011	m2-47	Uganda
Can181-9								
Chongqing-12-8	99.0							
Jiangsu-2011	100	99.0						
Sichuan-12-8	99.5	98.4	99.5					
Sichuan-12-12	100	99.0	100	99.5				
Guangdong-2011	90.7	90.1	90.7	90.1	90.7			
m2-47	90.9	90.4	90.9	90.4	90.9	100		
Uganda	80.3	80.3	80.3	79.8	80.3	80.2	81.8	
**RNA2-5′UTR**	Can181-9	Chongqing-12-8	Jiangsu-2011	Sichuan-12-8	Sichuan-12-12	Guangdong-2011	m2-47	Uganda
Can181-9								
Chongqing-12-8	100							
Jiangsu-2011	100	100						
Sichuan-12-8	99.5	99.5	99.5					
Sichuan-12-12	99.5	99.5	99.5	100				
Guangdong-2011	96.6	96.6	96.6	96.6	96.6			
m2-47	93.2	93.2	93.2	93.2	93.2	96.6		
Uganda	95.5	95.5	95.5	95.5	95.5	98.9	97.8	
**Hsp70h**	Can181-9	Chongqing-12-8	Jiangsu-2011	Sichuan-12-8	Sichuan-12-12	Guangdong-2011	m2-47	Uganda
Can181-9		99.1	98.9	99.1	99.3	90.4	90.4	90.6
Chongqing-12-8	99.0		99.1	98.6	98.7	90.1	90.1	90.3
Jiangsu-2011	98.9	99.5		98.4	98.6	90.1	90.1	90.3
Sichuan-12-8	99.4	98.8	98.9		99.1	90.1	90.1	90.3
Sichuan-12-12	99.4	98.8	98.9	99.6		90.1	90.1	90.3
Guangdong-2011	76.9	76.5	76.6	76.9	76.9		100	99.8
m2-47	77.0	76.5	76.7	76.9	77.0	99.9		99.8
Uganda	76.9	76.6	76.8	76.9	76.9	98.7	98.9	
**p60**	Can181-9	Chongqing-12-8	Jiangsu-2011	Sichuan-12-8	Sichuan-12-12	Guangdong-2011	m2-47	Uganda
Can181-9		97.5	97.3	97.5	97.5	78.8	78.8	79.5
Chongqing-12-8	98.7		99.4	98.5	98.5	79.7	79.7	80.5
Jiangsu-2011	98.3	99.4		98.6	98.6	79.3	79.3	80.1
Sichuan-12-8	99.0	98.7	99.0		99.2	79.5	79.5	80.3
Sichuan-12-12	98.9	98.7	98.9	99.7		79.5	79.5	80.3
Guangdong-2011	72.4	72.7	72.4	72.4	72.4		99.4	98.6
m2-47	72.5	72.8	72.5	72.5	72.4	99.8		98.8
Uganda	72.7	73.0	72.7	72.7	72.6	98.8	98.9	
**p8**	Can181-9	Chongqing-12-8	Jiangsu-2011	Sichuan-12-8	Sichuan-12-12	Guangdong-2011	m2-47	Uganda
Can181-9		98.6	95.9	97.3	98.6	68.5	68.5	68.5
Chongqing-12-8	98.2		97.3	98.6	100	69.9	69.9	69.9
Jiangsu-2011	98.2	98.2		95.9	97.3	68.5	68.5	68.5
Sichuan-12-8	98.6	98.6	98.6		98.6	69.9	69.9	69.9
Sichuan-12-12	98.6	98.6	98.6	99.1		69.9	69.9	69.9
Guangdong-2011	72.1	72.5	71.6	72.1	72.1		100	93.2
m2-47	72.1	72.5	71.6	72.1	72.1	100		93.2
Uganda	73.0	73.4	72.5	73.0	73.0	95.5	95.5	
**CP**	Can181-9	Chongqing-12-8	Jiangsu-2011	Sichuan-12-8	Sichuan-12-12	Guangdong-2011	m2-47	Uganda
Can181-9		99.2	99.2	99.6	98.4	79.8	79.4	77.8
Chongqing-12-8	99.5		98.4	99.6	98.4	79.4	79.0	77.4
Jiangsu-2011	99.4	98.8		98.8	97.7	80.2	79.8	78.2
Sichuan-12-8	99.6	99.6	99.0		98.8	79.8	79.4	77.8
Sichuan-12-12	99.4	99.4	98.7	99.5		78.6	78.2	76.7
Guangdong-2011	73.3	73.3	74.0	73.3	72.9		99.2	95.7
m2-47	73.3	73.3	74.0	73.3	72.9	99.7		96.5
Uganda	73.2	73.2	73.8	73.2	72.8	98.6	98.8	
**mCP**	Can181-9	Chongqing-12-8	Jiangsu-2011	Sichuan-12-8	Sichuan-12-12	Guangdong-2011	m2-47	Uganda
Can181-9		98.4	98.5	98.7	99.0	61.5	61.3	61.4
Chongqing-12-8	99.2		98.1	98.8	99.1	61.4	61.1	61.3
Jiangsu-2011	98.9	98.5		98.4	98.7	62.3	62.0	62.1
Sichuan-12-8	99.4	99.5	98.7		99.4	61.5	61.3	61.4
Sichuan-12-12	99.5	99.6	98.8	99.8		61.7	61.4	61.5
Guangdong-2011	63.5	63.2	63.6	63.3	63.4		99.1	98.7
m2-47	63.2	62.9	63.3	63.0	63.1	99.7		98.1
Uganda	63.4	63.1	63.6	63.1	63.2	98.4	98.2	
**p28**	Can181-9	Chongqing-12-8	Jiangsu-2011	Sichuan-12-8	Sichuan-12-12	Guangdong-2011	m2-47	Uganda
Can181-9		98.9	98.3	97.9	97.9	85.1	85.1	85.5
Chongqing-12-8	98.9		98.8	99.2	99.2	86.0	86.0	86.4
Jiangsu-2011	98.9	98.6		98.8	98.8	86.0	86.0	86.4
Sichuan-12-8	99.0	99.6	98.8		99.2	86.0	86.0	86.4
Sichuan-12-12	99.0	99.6	98.8	99.7		86.0	86.0	86.4
Guangdong-2011	77.4	77.6	77.9	77.5	77.5		99.2	97.9
m2-47	77.4	77.6	77.9	77.5	77.5	99.7		98.8
Uganda	77.9	78.2	78.5	78.1	78.1	97.9	98.2	
**RNA2-3′UTR**	Can181-9	Chongqing-12-8	Jiangsu-2011	Sichuan-12-8	Sichuan-12-12	Guangdong-2011	m2-47	Uganda
Can181-9								
Chongqing-12-8	99.5							
Jiangsu-2011	100	99.5						
Sichuan-12-8	99.5	100	99.5					
Sichuan-12-12	99.5	100	99.5	100				
Guangdong-2011	79.7	79.7	79.7	79.7	79.7			
m2-47	79.7	79.7	79.7	79.7	79.7	99.0		
Uganda	79.7	79.7	79.7	79.7	79.7	99.0	100	

Analysis of RNA1 3′ UTRs showed that this UTR, being 193 nt long (Table 3), is conserved between different isolates in WA strains and that its nucleotide identity is 98.4%–100% (Table 6). Isolates in the EA strain differed from each other significantly: the length of the 3′ UTR of isolate Uganda was 226 nt, that of isolate m2-47 was 187 nt long, and that of isolate Guangdong-2011 was 172 nt long (Table 3). The nt identity between Guangdong-2011 and m2-47 isolates was 100%, that between Uganda and m2-47 was 81.8%, and that between Guangdong-2011 and Uganda was 80.2% (Table 6).

Comparison of RNA2 5′UTRs showed that the WA strain length is 191 nt (Table 4) and that the nucleotide identity between different isolates is 99.5%–100% (Table 6). The nucleotide identity between the three isolates in the EA strain was 96.6%–98.9% (Table 6), the length of isolate Guangdong-2011 was 88 nt, and the lengths of the two other isolates were both 90 nt. Despite differences in the 5′ UTR lengths among WA and EA strains, the nucleotide sequences of these strains were rather conserved; nt identities between the eight isolates were 93.2%–100% (Table 6).

Comparison of RNA2 3′ UTRs suggested that the length of both WA and EA strains is 192 nt (Table 4) and that the nt identities between different isolates in the WA and EA strains exceed 99%. By contrast, the nt identity between WA and EA strains was 79.7%, which is rather low (Table 6).

Comparison of the 5′ and 3′ UTRs of RNA1 and RNA2 indicated that the nt identity of 5′UTRs between RNA1 and RNA2 is low (around 40%) whereas that of 3′UTRs of RNA1 and RNA2 is high (around 80%). The nt identity of 3′ UTRs of RNA1 and RNA2 of isolate Uganda was about 99%.

### Genome structure analysis and nucleotide/amino acid sequence comparison of proteins

Analysis of genome structures suggested that the RNA1 segment contained four ORFs: 1a (p227), RdRp, RNase3, and p7. The genome positions of these ORFs were listed in Table 5. Between protein p7 and the 3′UTR, the Uganda isolate contains a p22 ORF (located at 8606–9181 nt of the genome); none of the seven other isolates had the *p22* gene. Nucleotide and amino acid identities between these proteins are listed in Table 6.

Analysis of the nucleotide and amino acid identity of protein 1a (p227), RdRp, RNase3, and p7 encoded by eight isolates in RNA1 showed that the nucleotide identities of encoding proteins between the eight isolates are as follows: protein 1a (81.0%–99.6%), RdRp (87.4%–99.8%), RNase3 (82.7%–99.6%) and p7(75.0%–100%). The amino acid identities of encoding proteins between the eight isolates are as follows: protein 1a (87.2%–99.5%), RdRp (95.8%–99.8%), RNase3 (79.8%–99.6%), and p7 (60.0%–100%). The nt/aa identities between different isolates in the WA strain are as follows: protein 1a (98.7%–99.6%/98.6%–99.5%), RdRp (99.1%–99.8%/98.8%–99.6%), RNase3 (98.3%–99.6%/97.4%–99.6%), and p7 (98.2%–100%/96.4%–100%). The nt/aa identities between different isolates in the EA strain are as follows: protein 1a (98.3%–99.4%/97.6%–98.7%), RdRp (99.4%–99.6%/99.2%–99.8%), RNase3 (98.7%–99.4%/98.2%–99.1%), and p7 (98.3%–100%/98.2%–100%). The nt/aa identities between WA and EA strains are as follows: protein 1a (81.0%–81.3%/87.2%–87.6%), RdRp (87.4%–87.6%/95.8%–96.6%), RNase3 (82.7%–83.6%/79.8%–81.6%), and p7 (75.0%–78.0%/60.0%–63.6%). Analysis of RdRp protein sequences demonstrated that the 5′ end of isolate m2-47 is 16 aa longer than that of the other isolates. RNase3 sequence analysis showed that the ORF for RNase3 is 684 nt (228 aa) in all EA strain isolates but 687 nt (229 aa) in WA strain isolates. The 5′ end of the RNase3 ORF in EA strains was 1 aa longer than that of WA strains. P7 sequence analysis showed that the ORF for p7 is 171 nt (57 aa) in all EA strain isolates but 165 nt (55 aa) in WA strain isolates. The 3′ end of the p7 ORF in EA strains was 2 aa longer than that of WA strains.

Analysis of genomic structures indicated that the segment RNA2 contains at least six ORFs: Hsp70h, p60, p8, CP, mCP, and p28. The locations of these ORFs in the genome are listed in Table 4. The nucleotide and amino acid identities between these proteins are listed in Table 6.

Isolates Guangdong-2011, m2-47, and Uganda of the EA strain contained an ORF for p6 (named p6.1 in this study) in the upstream region of hsp70h, and the nt/aa identity of the p6.1 protein between these three isolates was 100%. Between p6.1 and hsp70h, similar to isolate m2-47, the Guangdong-2011 isolate contained one ORF for the p6 protein (named p6.2 in this study) more than isolate Uganda; the nt and aa identities of this protein were 98.7% and 96.2%, respectively. Similar to Can181-9, isolates Jiangsu-2011, Sichuan-12-8, Sichuan-12-12, and Chongqing-12-8 of the WA strain contained three ORFs in the upstream region of hsp70h, namely p5.2, p5, and p5.1. The nt and aa identities of the five isolates are as follows: p5.2 (97.8%–100% and 100%), p5 (97.0%–100% and 90.9%–100%), and p5.1 (95.3%–100% and 90.5%–100%).

Analysis showed that the nucleotide identities of the encoded proteins between the eight isolates are as follows: Hsp70h (76.5%–99.9%), p60 (72.4%–99.8%), p8 (72.1%–100%), CP (72.8%–99.7%), mCP (62.9%–99.8%), and p28 (77.4%–99.7%). The aa identities of these proteins are as follows: Hsp70h (90.1%–100%), p60 (78.8%–99.4%), p8 (68.5%–100%), CP (76.7%–99.6%), mCP (61.1%–99.4%), and p28 (85.1%–99.2%). The nt/aa identities between different isolates of WA strain are as follows: Hsp70h (98.8%–99.6%/98.4%–99.3%), p60 (98.3%–99.7%/97.3%–99.4%), p8 (98.2%–99.1%/95.9%–100%), CP (98.7%–99.6%/97.7%–99.6%), mCP (98.5%–99.8%/98.1%–99.4%), and p28 (98.6%–99.7%/97.9%–99.2%). The nt/aa identities between different isolates within the EA strain are as follows: Hsp70h (98.7%–99.9%/99.8%–100%), p60 (98.8%–99.8%/98.6%–99.4%), p8 (95.5%–100%/93.2%–100%), CP (98.6%–99.7%/95.7%–99.2%), mCP (98.2%–99.7%/98.1%–99.1%), and p28 (97.9%–99.7%/97.9%–99.2%). The nt/aa identities between the WA and EA strains are as follows: Hsp70h (76.5%–77.0%/90.1%–90.6%), p60 (72.4%–73.0%/78.8%–80.5%), p8 (72.1%–73.4%/68.5%–69.9%), CP (72.8%–74.0%/76.7%–80.2%), mCP (62.9%–63.6%/61.1%–62.3%), and p28 (77.4%–78.5%/85.1%–86.4%). CP protein sequence analysis also showed that isolate Uganda has one methionine residue more than the seven other isolates in locus 231.

### Absence of the *p22* gene in RNA1

Genome structure analysis showed that the *p22* gene was not present in any of the aforementioned isolates from China. To investigate whether or not *p22* is present in other Chinese regions, the primers RdRp-F and SVV-R3 were used to amplify the region from the *RdRp* gene to the 3′-UTR of RNA1; these primers were designed according to the reference [28]. Sweet potato samples infected by SPCSV were collected from Jiangsu, Zhejiang, Anhui, and Chongqing, China. Using RT-PCR, the partial RNA1 sequences from four different samples were obtained (relevant data, such as name, strain assignment, geographic origin, segment length, reference, and GenBank accession numbers, are listed in Table 1). Sequence analysis indicated that Zhejiang-11-4 shares nucleotide identities of 99.6% with Guangdong-2011 and 84.4% with isolate Jiangsu-2011. Isolates Jiangsu-11-19, Anhui-11-2, and Chongqing-11-8 shared nucleotide identities of 84.4% with isolate Guangdong-2011 and 99.4%–99.6% with isolate Jiangsu-2011. Overall, genome organization analysis showed that *p22* is missing in all nine isolates from China.

**Figure 3:**
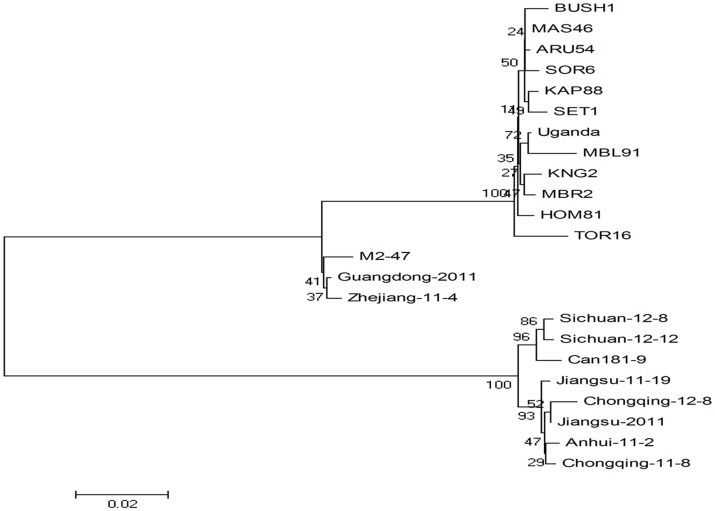
Phylogenetic tree based on partial sequences of RNA1. Phylogenetic tree illustrating relationships between isolates obtained from China and representative isolates of sweet potato chlorotic stunt virus (SPCSV) deposited in GenBank. Neighbor-joining trees were constructed via the maximum composite likelihood substitution model using MEGA (version 4.0). Numbers at branches represent bootstrap values of 1000 replicates. The scale bar shows the number of nucleotide substitutions per site.

The nt sequences of 9 SPCSV isolates characterized in this study and 14 isolates deposited in GenBank were subjected to phylogenetic analysis, including the previously characterized WA strain Can181-9 isolate from Spain, EA strain m2-47 isolate from Peru, and 12 EA strain isolates from Uganda. Phylogenetic analysis (Figure 3) of partial genomic sequences of RNA1 was used to assign isolates of SPCSV from China to the two relatively distantly related strains EA and WA. Isolates Zhejiang-11-4 and Guangdong-2011 in this study belonged to the EA strain. Isolates Jiangsu-11-19, Anhui-11-2, Chongqing-11-8, Jiangsu-2011, Sichuan-12-8, Sichuan-12-12, and Chongqing-12-8 belonged to the WA strain.

### Molecular variability and phylogenetic analysis of the *cp* gene in WA strains

To study the genetic variation of SPCSV in different Chinese regions, we used the nucleotide sequence of the *cp* gene of the SPCSV WA strain in GenBank (GenBank accession number FJ807785) to design primers named CP-F and CP-R to amplify the *cp* gene of SPCSV WA isolates from different regions. A total of 20 *cp* gene sequences from 14 Chinese provinces or cities were obtained (name, strain assignment, geographic origin, segment length, reference, and GenBank accession numbers are listed in Table 1). Sequencing results suggested that the *cp* gene sequence length of 20 isolates is 774 bp, as expected.

**Figure 4:**
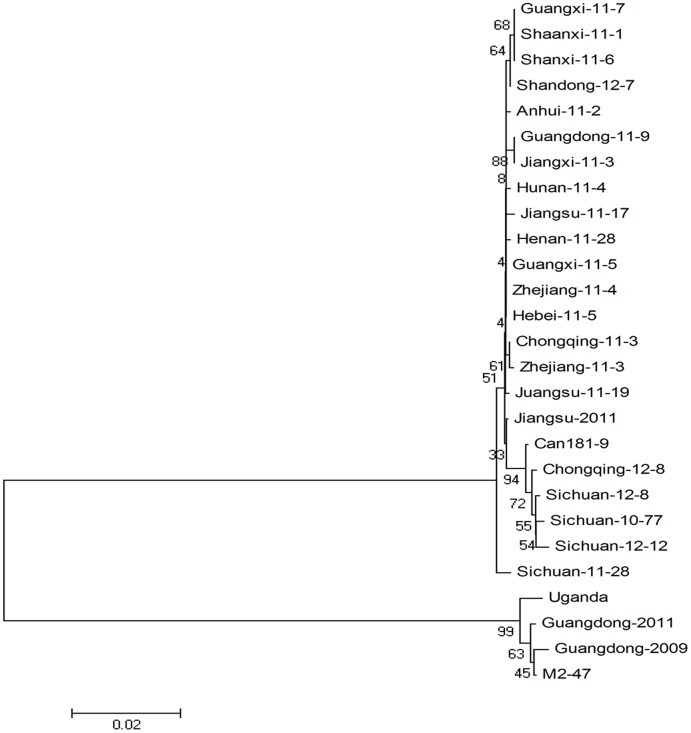
Phylogenetic tree based on CP gene sequences. Phylogenetic tree illustrating relationships between the CP genes of isolates obtained from China and representative isolates of sweet potato chlorotic stunt virus (SPCSV) deposited in GenBank. Neighbor-joining trees were constructed via the maximum composite likelihood substitution model using MEGA (version 4.0). The bar in this figure represents 0.02 Kimura nucleotide unites. Numbers at branches represent bootstrap values of 1000 replicates.

Sequence alignment and phylogenetic analysis using the neighbor-joining method were performed with MEGA software (version 4.0). Nucleotide and amino acid sequence identity analysis of 25 isolates acquired in this research showed that, except for isolate Guangdong-2011 (EA strain), SPCSV isolates belonging to the WA strain exhibit 98.4%–100% nt sequence identity and 97.7%–100% deduced aa sequence identity. These findings demonstrate that isolates of the SPCSV WA strain from China show nearly identical. A phylogenetic tree was constructed (Figure 4) based on CP sequences to illustrate the probable genetic relationships between the 25 isolates and Guangdong-2009, m2-47, Uganda, and Can181-9. Results showed that Chinese SPCSV isolates could be divided into two branches. Except for those obtained from the Guangdong region, SPCSV isolates from other Chinese regions belonged to the WA strain. This finding indicates that at least two different strains may be observed among Chinese SPCSV isolates and that the WA strain is more prevalent than the EA strain.

Analysis of the phylogenetic tree based on partial RNA1 sequences showed that Zhejiang-11-4 from Zhejiang Province belongs to the EA strain. However, when the tree was constructed using the CP gene, the isolate appeared to belong to the WA strain. This finding suggests that Zhejiang-11-4 occurs through co-infection by WA and EA.

This study reveals that nucleotide and amino acid identities are highly conserved between different isolates of Chinese strains and that the WA strain is distributed more extensively than the EA strain.

## Discussion

The complete genomic sequences of five different SPCSV isolates were acquired from main sweet potato production areas in China by RT-PCR and 5′ and 3′-RACE using virus-transmitting vector whitefly as a material. Guangdong-2011, which was isolated from Guangdong Province, belongs to the EA strain, whereas Jiangsu-2011, Sichuan-12-8, Sichuan-12-8, and Chongqing-12-8, which were isolated from Jiangsu, Sichuan, and Chongqing, belong to the WA strain. To the best of our knowledge, this study is the first to report the complete genomic sequences of Chinese SPCSV isolates as well as the complete SPCSV genomic sequence acquired from whitefly.

Factors such as low titers of SPCSV, heterogeneous distribution of the virus in sweet potato, and phenol and polysaccharide enrichment in sweet potato leaves [40], [41] significantly decrease the quality of extracted RNA from sweet potato leaves and the amplification efficiency of conventional RT-PCR. SPCSV usually co-infects sweet potato with other viruses in the field; thus SPCSV virions are difficult to separate and purify. These factors severely restrict SPCSV genome sequencing [22], [32]–[34]. Only three SPCSV complete genomic sequences can be accessed via GenBank today, and only one complete sequence of SPCSV WA strain is available [11], [29], [35]. In the present study, the SPCSV titer in whitefly was found to be significantly higher than that in sweet potato and *I. setosa*. This feature of the virus-transmitting vector whitefly is advantageous for acquiring the complete SPCSV genomic sequence. In addition, using viruliferous whitefly as an experimental material can preclude the interference of other viruses, phenols, and polysaccharides in sweet potato leaves. Our research provides a simplified and convenient method for cloning SPCSV genomic sequences.

The complete genomic sequences of the five isolates from different Chinese areas were used to analyze molecular variations and phylogenesis compared with the genomic sequences of three isolates obtained from GenBank. Analysis of the nucleotide identity of complete genomic sequences indicated that the nt identities in SPCSV strain are highly conserved and significant differences were found between WA and EA strains. Analyses of nt and aa identities based on the *cp* gene and partial RNA1 sequences are consistent with aforementioned results. High conservation was observed between different isolates in the same strain, but significant differences were found between WA and EA strains, which are inferred to be caused by the short time since SPCSV was transmitted into China, so the virus shows only limited genetic variability. This result is consistent with previous reports [3], [27], [28], [36]. Tugume [36] analyzed the genetic variability of *p7*, *RNase3*, and *p22* genes of different SPCSV isolates infecting sweet potato and wild species in Uganda and suggested that the three genes show only limited genetic variability among strains. The EA and WA strain isolates showed nt (aa) sequence identities of 83.8%–84.3% (81.6%–82.5%) for RNase3 and 76.2%–79.8% (60.7%–66.7%) for p7 [36]. The nt and deduced aa sequence identities of *RNase3*, *p7* and *hsp70h* of EA strain isolates Ug, Tug2, Unj2, Mis1, m2-47, and WA strain isolate Is were analyzed. The nt and aa sequences of *RNase3* and *hsp70h* in the EA strain isolates were nearly identical but the nt and aa of *p7* showed more variation [28]. We previously analyzed the genetic variability of partial *hsp70h* gene of different SPCSV isolates in China, and our results demonstrated that the *hsp70* gene sequences of the same strain group display a high degree of conservation and that strain group WA has a wider geographic distribution in China than the EA strain [27].

The genome organization of SPCSV shares many similarities with other criniviruses [10]. However, the genome of SPCSV possesses unique features particularly concerning the gene content of RNA1. Downstream from the ORF for the replicase, RNA1 contains ORFs for Class 1 RNase III enzyme, a putative hydrophobic protein (p7), and a 22 kDa protein that shows no significant similarity to known proteins from any organism [11], [28], [42]. The 3′-proximal part of RNA1 constitutes an interesting genomic region for study owing to its unique gene functions. RNase3 contains a single endoribonuclease domain and a dsRNA-binding domain [43], [44]. RNase3 inhibits posttranscriptional gene silencing and exerts a key function in the development of severe diseases in sweet potato plants co-infected with other viruses [42], [43]. The *p22* gene was identified as a SPCSV RNA silencing suppressor and it could suppress silencing induced by dsRNA [42]. Similar activities were exhibited by homologs of *p22* encoded by other members of the family *Closteroviridae*. Cañizares identified *Tomato chlorosis virus* (ToCV) RNA1-encoded p22 protein as an effective silencing suppressor by using an agrobacterium co-infiltration assay. ToCV p22 suppressed local RNA silencing induced either by sense RNA or dsRNA very efficiently but did not interfere with short or long-distance systemic spread of silencing [45]. Data showed that the *Beet yellow virus* p21, *Beet yellow stunt virus* p22, *Citrus tristeza virus* p20, and *Grapevine leafroll-associated virus-2* p24, which are homologs of p22, are silencing suppressors [46]–[48]. Recent studies have revealed that many SPCSV isolates lacking *p22* still synergize with unrelated viruses [18], [28], [43], which indicates that p22 is dispensable for synergy between SPCSV and other viruses. While *p22* is a pathogenicity enhancer of SPCSV, co-infection of SPFMV with SPCSV isolates containing *p22* causes more severe symptoms than co-infection with SPCSV isolates lacking *p22* in the indicator plant *I. setosa* [28]. The *p22* gene is present only in Ugandan isolates of SPCSV, and SPCSV isolates from other areas do not contain the *p22* gene [36]. The partial RNA1 sequences of nine isolates from different Chinese regions were determined in this study, and no isolate (neither EA nor WA strains) containing the *p22* gene was observed in our research.

A novel virus isolate related to viruses in genus *Crinivirus* carrying predicted ORFs for proteins homologous to the RNase3 and p7 of SPCSV was detected and designated as KML33b. The sequences of KML33b were highly divergent from SPCSV isolates and showed <60% sequence identities for both nt and aa [36]. According to the species demarcation criteria from the Ninth Report of the International Committee on Taxonomy of Viruses (ICTV), the criteria for demarcating species in the genus *Crinivirus* are: (a) genome structure and organization (number and relative location of the ORFs) and (b) amino acid sequences of relevant gene products (polymerase, CP, Hsp70h) differing by more than 25% [49]. In the present study, comparison of RNA1 and RNA2 between WA and EA strains showed that the nucleotide identities exhibit significant differences between strains. Genomic structure analysis suggested that the quantity of proteins encoded by the RNA2 segment between SPCSV WA and EA strains differ. Before *hsp70h*, WA encodes three but EA encodes one or two hypothetical proteins. Comparison of the 3′ and 5′ UTRs of the genome shows that the lengths of RNA1 3′UTR and RNA2 5′UTR are different between WA and EA strains. The nucleotide identity of the RNA1 3′UTR and RNA2 5′UTR between WA and EA strains is low. Although the RNA1 5′ and RNA2 3′UTRs of the WA and EA strains showed the same length, their nucleotide identity was low. Comparison of aa identities suggested that proteins with an aa identity <75% between the WA and EA strains include p7 in RNA1, p8, and mCP in RNA2. Tairo previously suggested that EA and WA of SPCSV may belong to different species in the genus *Crinivirus* [3]. As mentioned above, the low sequence identity and differences in genomic structures between the EA and WA strains of SPCSV support this proposal. However, as the biological and serological relationships between the strains remain incompletely understood, a systematic assessment of these differences should be given the top priority for future research.

## Materials and Methods

### Ethics statement

Samples were collected from private land with the owner's permission. No specific permissions were required for sampling from any other location. Field studies did not involve endangered or protected species.

### Collection of virus isolates

From 2010 to 2012, five sweet potato vine cuttings were collected from the main sweet potato-producing areas (Jiangsu, Guangdong, Sichuan Provinces, and Chongqing City) of China (Table 1) and grown in an insect-proof greenhouse. These cuttings were proven to be infected with SPCSV by nitrocellulose membrane (NCM)-ELISA and RT-PCR. The SPCSV-infected sweet potato was placed in an insect cage to feed the whiteflies. Non-viruliferous whiteflies were separately fed in SPCSV-infected sweet potato plants; here, whiteflies that had been fed for more than 3 d were considered viruliferous. Adult viruliferous whiteflies were collected and quick-frozen in liquid nitrogen, and then stored at −70°C to amplify complete genomic sequence.

From 2010 to 2012, a total of 20 vine cuttings were collected from the main sweet potato-producing areas in 14 provinces of China (Table 1). *I. setosa* was inoculated by side-grafting with infected sweet potato scions and grown in an insect-proof greenhouse (temperature, 25–30°C; relative humidity, 70%) under natural daylight. *I. setosa*-infecting SPCSV were used to clone the *cp* gene and partial RNA1 sequences.

### Design and synthesis of primers used in this study

The primers used in this study are shown in Table 2. Primers for amplification of the RNA1 and RNA2 regions of WA strains were designed according to the sequences of SPCSV and deposited in GenBank (GenBank accession numbers FJ807784 and FJ807785). The primers were named WA-RNA1-P1, WA-RNA1-P2989, WA-RNA1-P2420, WA-RNA1-P5596, WA-RNA1-P4410, WA-RNA1-P8637, WA-RNA2-P53, WA-RNA2-P5599, WA-RNA2-P4440, and WA-RNA2-P8223. Primers for amplification of the RNA1 and RNA2 regions of EA strains were designed according to the sequences of SPCSV and deposited in GenBank (GenBank accession numbers HQ291259, HQ291260, AJ428554, and AJ428555). The primers were named EA-RNA1-P15, EA-RNA1-P4800, EA-RNA1-P3680, EA-RNA1-P8410, EA-RNA2-P1, EA-RNA2-P4910, EA-RNA2-P3710, and EA-RNA2-P8130. Primers for amplification of the *cp* gene were designed according to the sequences of SPCSV and deposited in GenBank (GenBank accession number FJ807785). These primers were named CP-F and CP-R. Primers for amplification of the 3′ genomic region of RNA1 were synthesized according to the reference [28] and named RdRp-F and SVV-R3.

### Serological detection of SPCSV

SPCSV was detected using an NCM-ELISA kit from the International Potato Center (CIP). Briefly, 150 mg of leaf material was ground in a mortar with 1 mL of extraction buffer (1 M Tris-HCl containing 0.2% Na_2_SO_3_). The homogenate was transferred to a 1.5 mL Eppendorf tube and spun at 6000×*g* for 5 min. Aliquots of the supernatant (20 µL) were placed on membranes. The membranes were blocked with blocking solution at room temperature and then incubated for 60 min. After washing twice for 3 min each time, the membranes were incubated in virus-specific antibodies to the SPCSV coat protein (provided by CIP) at 4°C overnight. After washing twice for 3 min each time, the membranes were incubated in the conjugated anti-SPCSV antibody at room temperature for 1 h and then washed again twice for 3 min each time. The color reaction was developed using NBT/BCIP as the substrate. Color development was ceased by discarding the substrate solution and immersing the membranes in tap water. NCMs were washed in distilled water for 10 min.

### RNA isolation

Total RNA was isolated from 100 mg of viruliferous whiteflies or *I. setosa* leaves as templates using the Total Plant RNA Extraction Miniprep System (Sangon, Shanghai, China). The amount and quality of the RNA were verified using agarose gel electrophoresis.

### RT-PCR detection of SPCSV

RNA was used for reverse transcription using Moloney murine leukemia virus (M-MLV) reverse transcriptase (TaKaRa, Shiga, Japan) according to the manufacturer's instructions. Synthesized cDNA was amplified using Ex *Taq* DNA polymerase (TaKaRa). The partial sequence of *hsp70h* gene was amplified to confirm samples infected SPCSV. Degenerate primers for amplifying the *hsp70h* gene were designed according to the sequences of the SPCSV WA and EA strains and deposited in GenBank. These primers were respectively named Hsp70h-F and Hsp70h-R (Table 2).

### Cloning and sequence analysis

RNA was used for reverse transcription using M-MLV reverse transcriptase (TaKaRa) according to the manufacturer's instructions. Synthesized cDNA was amplified using LA *Taq* DNA polymerase (TaKaRa). Genome RNA1 or RNA2 sequences of the WA strain were acquired by two or three overlapping RT-PCRs, whereas genome RNA1 or RNA2 sequences of the EA strain were obtained by two overlapping RT-PCRs. All PCR conditions in this study are shown in Table 2. Rapid amplification of cDNA ends (RACE) was used to determine the 5′ and 3′ ends of the viral genomic segments RNA1 and RNA2. 5′ and 3′ RACE was conducted by TaKaRa. The *cp* gene and the partial sequence of RNA1 obtained from the 3′-promixmal region of the RdRp gene to the middle of the 3′UTR were determined by cloning and sequencing obtained amplicons by standard RT-PCR.

The amplified products were purified from agarose gels using an AxyPrep DNA Gel Extraction Kit (Axygen, Hangzhou, China) and cloned into the PMD19-T vector (TaKaRa). Recombinant plasmids were transformed into *Escherichia coli* strain TG1 competent cells, purified using Plasmid Miniprep Kits (Bioteke, Beijing, China), and sequenced by Sangon Biotech Company (Sangon, Shanghai, China). Sequencing was conducted in both directions for each of the two independent amplicons of all isolates.

The sequences obtained were compared by BLAST search with the existing sequences in the NCBI database. Sequences were analyzed using DNAMAN Version 6.0. SPCSV Can181-9, m2-47 and Uganda isolates were used to explore the genome organization of the SPCSV WA and EA strains in China. Phylogenetic and molecular evolutionary analyses were conducted using MEGA version 4.0 [50]. Multiple alignments of protein-coding sequences were obtained using the default options in Clustal W [51]. Phylogenetic trees were constructed based on the aligned protein-coding sequences using the neighbor-joining method. The statistical significance of tree branching was tested by performing 1000 bootstrap replications.
